# Diabetes knowledge among non-diabetic hypertensive patients in Calabar, Nigeria

**DOI:** 10.11604/pamj.2020.36.198.20522

**Published:** 2020-07-20

**Authors:** Ofem Egbe Enang, Ogban Ezukwa Omoronyia, Agam Ebaji Ayuk, Kenneth Nnachetam Nwafor, Anette Oshioagiemhe Legogie

**Affiliations:** 1 Department of Internal Medicine, University of Calabar, Calabar, Nigeria,; 2 Department of Community Medicine, University of Calabar, Calabar, Nigeria,; 3 Department of Family Medicine, University of Calabar, Calabar, Nigeria

**Keywords:** Diabetes mellitus, knowledge, hypertensive, prevention, Nigeria

## Abstract

**Introduction:**

among hypertensive patients, the comorbidity of diabetes is not uncommon. Yet, little is known about diabetes prevention among non-diabetic hypertensive patients. This study sought to assess such patients' knowledge about diabetes and its risk factors.

**Methods:**

a cross-sectional descriptive study design and random sampling were used to recruit non-diabetic hypertensive patients from University of Calabar Teaching Hospital. A pretested 33-item questionnaire was used to assess various aspects of diabetes knowledge. Participants' alcohol consumption, smoking habits, physical activity, and fresh fruit consumption were also assessed. The p-value was set to 0.05.

**Results:**

of 212 respondents with a mean age of 45.5 ± 10.8 years, approximately half (49.1%) had inadequate knowledge of diabetes. Most participants demonstrated poor knowledge of diabetes' clinical features (81.1%) and complications (59.4%), while fewer participants showed poor knowledge of causes and risk factors (24.5%) and diabetes management (40.6%). Older subjects, those in the wards, non-drinkers, physically active people, and those who frequently consumed fresh fruit had a significantly greater understanding of diabetes symptoms and complications (p<0.05).

**Conclusion:**

hypertensive patients' diabetes knowledge is generally suboptimal, with greater knowledge deficiencies being apparent in specific areas. More strategic health education initiatives are required, about minimizing the risk of developing diabetes comorbidities.

## Introduction

Diabetes mellitus is a potentially life-threatening chronic disease and a significant public health concern [1]. Currently, it is estimated that 451 million people worldwide have diabetes, many of whom are undiagnosed and from low to middle-income countries [2]. Obesity and other risk factors for diabetes onset and progression are also rapidly on the rise [1]. Fortunately, diabetes can be prevented or delayed through the regular practice of preventive measures [3], but diabetes knowledge has been found to be inadequate in many developing countries in which the disease is becoming more prevalent [4]. Poor knowledge of diabetes, especially among high risk individuals, may be contributing to the high burden of the disease in sub-Saharan Africa [1,3]. Non-diabetic hypertensive patients are considered a high-risk group for diabetes. Both hypertension and diabetes have many common risk factors, including physical inactivity and inappropriate dietary intake [5,6]. Reports of hypertension diagnoses at earlier ages and decades, as well as the later onset of most diabetes mellitus cases among adults, may be contributing to the masking of diabetes in a significant portion of at-risk hypertensive patients [7]. Additionally, long-term use of certain antihypertensives, such as diuretics and non-dihydropyridine calcium channel blockers, have been associated with an increased risk of diabetes among hypertensive patients [8,9]. As such, many patients with hypertension are prone to developing diabetes, especially if preventive measures are not taken.

Previous studies have indicated key risk-related lifestyle differences between diabetic and non-diabetic hypertensive patients. For instance, in a cohort study of hypertensive patients, the incidence rate of new-onset diabetes (NOD) was significantly higher among obese subjects compared to non-obese subjects (100.7 vs. 63.8 per 1,000 people, respectively) [10]. Hypertensive patients´ consumption of table salt has been linked with a twofold increased risk of NOD [11]. Hypertensive patients who develop diabetes also experience greater difficulty in controlling their systemic blood pressure compared to those who remain non-diabetic [12]. Hypertensive patients who develop diabetes, experience an earlier onset and more rapid progression of end-organ damage, mainly due to endothelial dysfunction [13]. Such comorbidity can have untold impacts on overall disease progression, prognosis, quality of life, and cost of healthcare [14]. Hypertensive patients must therefore know how to prevent or delay the onset and progression of diabetes. Non-hypertensive patients´ knowledge of these factors may be useful towards their compliance with lifestyle modifications meant to reduce the risk of diabetes and its complications. Unfortunately, most studies about diabetes knowledge are conducted among individuals in the general population or among patients who are already diabetic; high risk groups, including hypertensive patients, are often neglected in research [15,16]. There is thus a paucity of literature detailing effective health education and other intervention strategies that are more specific to non-diabetic hypertensive patients [17]. Consequently, this study aimed to determine diabetes knowledge among non-diabetic hypertensive patients in a developing sub-Saharan African setting.

## Methods

This study followed a descriptive cross-sectional design and recruited non-diabetic hypertensive patients from University of Calabar Teaching Hospital (UCTH), using a systematic random sampling method. Outpatient and medical ward registers were used as a sampling frame for recruitment. Known diabetic hypertensive patients were excluded from participation. In addition, outpatients who had only visited the clinic once, as well as patients that had spent fewer than 24 hours in the ward, were excluded from the study. Quantitative data was obtained using a 33-item interviewer-administered structured questionnaire. The instrument was a validated tool that has been tested in diverse global settings [18,19] and was pretested in the study setting before using it for data collection. Four components of diabetes knowledge were defined: causes and risk factors, symptoms, complications and management. Sociodemographic and behavioural characteristics, including alcohol consumption, smoking, physical activity and consumption of fresh fruit, were also assessed. Data collectors were trained and supervised by the researchers. SPSS version 21.0 was used to enter and analyse data. The proportion of correct and incorrect responses for each item assessed was presented per knowledge component. Each correct response contributed to the total scores for each component; scores of 50% and above were considered good or satisfactory. Mean scores were obtained and compared between sociodemographic and behavioural group characteristics using an independent t-test and analysis of variance (ANOVA). The p-value was set to 0.05. Consent from respondents and approval from the UCTH research ethics committee were obtained before the study commenced.

## Results

Complete data was obtained from 212 respondents with a mean age of 45.5 ± 10.8 years, ranging from 26 to 67 years old, with a male/female ratio of 1: 0.62. Most respondents were 40 years and older (69.8%), married (84.0%), civil servants (51.9%), had secondary school education (91.5%) and were recruited from a medical outpatient clinic (91.5%) (Table 1). 76 respondents (35.5%) consumed alcohol, while four (1.9%) smoked cigarettes (Table 2). Approximately one-tenth (10.4%) engaged in active physical exercise and a quarter (25.5%) consumed fresh fruit, each three or more times a week. Many incorrect responses were submitted regarding the assumed consumption of sweet foods as a cause of diabetes (80.2%), diabetes caused by kidney failure (81.1%), the kidney as a source of insulin (81.1%) and shaking and sweating as a sign of high blood sugar (71.2%) (Table 3). Other answers expressed thoughts that frequent urination signaled low sugar (71.7%), regular exercise increased need for insulin and medications (73.6%) and special foods were a primary component of the diabetic diet (78.3%) (Table 3). Approximately three-quarters of respondents (75.5%) had good knowledge about diabetes causes and risk factors (Table 4), but less than one-fifth (18.9%) demonstrated satisfactory knowledge of its clinical features. 40.6% and 59.4% of respondents showed awareness of diabetes complications and diabetes management, respectively.

**Table 1:** sociodemographic characteristics of respondents (N=212)

Variable	Frequency	Percentage
**Gender**		
Male	131	61.8
Female	81	38.2
**Age group** (in years)		
<30	14	6.6
30-39	50	23.6
40-49	71	33.4
50-59	47	22.2
≥60	30	14.2
**Marital status**		
Married	178	84.0
Single	28	13.2
Divorced/separated	2	0.9
Widowed	4	1.9
**Educational level**		
None	4	1.9
Primary	14	6.6
Secondary	72	34.0
Tertiary	122	57.5
**Occupation**		
Civil servant	110	51.9
Public servant	34	16.0
Business/trader	60	28.3
Others	8	3.8
**Source of respondent**		
Ward	18	8.5
Clinic	194	91.5

**Table 2:** behavioural risk and perception of health status (N=212)

Variable	Frequency	Percentage
**Consume alcohol**		
Yes	76	35.8
No	136	64.2
**Smoke**		
Yes	4	1.9
No	208	98.1
Physical exercise^*^		
Never	24	11.3
Occasionally (less than once a week)	72	34.0
Once or twice weekly	94	44.3
Three or more times weekly	22	10.4
Consume fresh fruits^**^		
Never	18	8.5
Occasionally (less than once a week)	56	26.4
Once or twice weekly	84	39.6
Three or more times weekly	54	25.5

^*^ exercise considered adequate if it occurs for at least 30 minutes (for active) and 50 minutes for passive ^**^ fresh fruit consumption considered adequate if 3 to 5 servings are consumed within 24 hours

**Table 3:** frequency distribution of knowledge of diabetes mellitus (N=212)

Variable		Correct response n (%)	Incorrect response n (%)
**Causes, risk factors, and nature of diabetes**			
1	Eating too much sugar and other sweet foods is a cause of diabetes.	42 (19.8)	170 (80.2)
2	The usual cause of diabetes is lack of effective insulin in the body.	122 (57.2)	90 (42.5)
3	Diabetes is caused by failure of the kidneys to keep sugar out of the urine	40 (18.9)	172 (81.1)
4	Kidneys produce insulin.	40 (18.9)	172 (81.1)
5	If I am diabetic, my children have a higher chance of being diabetic	98 (46.2)	114 (53.8)
6	There are two main types of diabetes: Type 1 and Type 2	139 (65.1)	74 (34.9)
7	Is diabetes a serious national health issue in Nigeria?	156 (73.6)	56 (26.4)
8	Diabetes mellitus is a disease of the rich	134 (63.2)	78 (36.8)
9	Diabetes is linked to being obese or being overweight	130 (61.3)	82 (38.7)
10	Regular physical activity accompanied by food rich in fiber, whole grain-based diet rich in vegetables and fruits is forsaken by many people	150 (70.8)	62 (29.5)
11	Cakes, ice-creams and other ‘sweet’ foods should be eaten at the end of a meal	124 (58.5)	88 (41.5)
**Symptoms, signs & diagnosis of diabetes**			
12	In untreated diabetes, the amount of sugar in the blood usually increases	144 (67.9)	68 (32.1)
13	A fasting blood sugar level of 210 is too high.	146 (68.9)	66 (31.1)
14	The best way to check my diabetes is by testing my urine.	76 (35.80)	136 (64.2)
15	Diabetes can cause loss of feeling in my hands, fingers and feet.	74 (34.9)	138 (65.1)
16	Shaking and sweating are signs of high blood sugar.	52 (24.5)	160 (75.5)
17	Frequent urination and thirst are signs of low blood sugar.	60 (28.3)	152 (71.7)
**Complications of diabetes**			
18	Diabetes often causes poor blood circulation.	78 (36.8)	134 (63.2)
19	Diabetes can damage my kidneys.	122 (57.5)	90 (46.5)
20	An insulin reaction is caused by eating too much food.	74 (34.9)	138 (65.1)
21	Cuts and abrasions on diabetics heal more slowly.	148 (69.8)	64 (30.2)
22	People with pre-diabetes (high fasting blood glucose), are at 50% higher risk of heart disease and stroke than people who do not have pre-diabetes	124 (58.5)	88 (41.5)
23	Diabetes increases risk of blindness	78 (36.8)	134 (63.2)
**Treatment & management of diabetes**			
24	Diabetes mellitus can be cured.	112 (52.8)	100 (47.2)
25	Traditional healers can cure diabetes using herbs	114 (53.8)	98 (46.2)
26	Regular exercise will increase the need for insulin or other diabetic medication	56 (26.4)	156 (73.6)
27	Medication is more important than diet and exercise to control my diabetes	68 (32.1)	144 (67.9)
28	Diabetics should take extra care when cutting their toenails.	140 (66.0)	72 (34.0)
29	A person with diabetes should cleanse a cut with iodine and alcohol.	116 (54.7)	96 (45.3)
30	The way I prepare my food is as important as the foods I eat	162 (76.4)	50 (23.6)
31	Tight elastic hose or socks are not bad for diabetics.	104 (49.1)	108 (50.9)
32	A diabetic diet consists mostly of special foods.	46 (21.7))	166 (78.3)
33	When obesity and lack of exercise are detected earlier, diabetes can be prevented.	146 (68.9)	66 (31.1)

**Table 4:** distribution of sub-scores for knowledge of diabetes (N=212)

Variable	Frequency	Percentage
**Section 1** (Causes, risk factors, and nature of diabetes score)		
Poor (≤5)	52	24.5
Good (≥5)	160	75.5
Mean ± SD (range)	5.54 ± 1.87 (0-10)	
**Section 2** (Symptoms, signs & diagnosis of diabetes score)		
Poor (≤3)	172	81.1
Good (>3)	40	18.9
Mean ± SD (range)	2.60 ± 1.23 (0-6)	
**Section 3** (Complications of diabetes score)		
Poor (≤3)	126	59.4
Good (>3)	86	40.6
Mean ± SD (range)	2.94 ± 1.53 (0-6)	
**Section 4** (Treatment & management of diabetes score)		
Poor (≤5)	86	40.6
Good (≥5)	126	59.4
Mean ± SD (range)	5.02 ± 1.8	(1-9)
**Total score**		
≤50% (inadequate knowledge)	108	50.9
≥50% (adequate knowledge)	104	49.1
Mean % ± SD (range)	48.8 ± 13.2	(12.1-78.8)

The mean scores for all components of diabetes knowledge were significantly higher among males, but statistical significance was found only in the results about diabetes complications (p<0.05, Table 5). The mean score on knowledge of complications was significantly higher among respondents 50 years or older compared to other age groups (p<0.05). Married respondents had a significantly higher mean score on knowledge of diabetes symptoms and complications compared to their unmarried counterparts (p<0.05). Compared to civil servants, public servants and businesspeople/traders had a significantly higher mean score for all components of their assessed diabetes knowledge (p=0.00). Respondents from the wards, with the exception of their knowledge about diabetes causes, had a significantly higher mean score on overall diabetes knowledge compared to those from the clinic (p=0.00). Compared to non-drinkers, alcohol consumers had a significantly lower mean score on diabetes symptom and complication knowledge (p=0.00, Table 6). However, there was no significant difference in the mean scores on all components of diabetes knowledge between smokers and non-smokers. Except for knowledge of symptoms, the mean score on all other components was higher among respondents that exercised three or more times weekly compared to other groups, but statistical significance was found only for knowledge about complications (p<0.05). The mean score on symptom knowledge and prevention was significantly lower among those who ate fruit less frequently (p<0.05).

**Table 5:** relationship between sociodemographic factors and knowledge of diabetes (N=212)

Variable	Total score Mean ± SD	Cause of DM Mean ± SD	Symptoms Mean ± SD	Complications Mean ± SD	Management Mean ± SD
**Gender**					
Male	49.0 ± 12.9	5.55 ± 1.76	2.63 ± 1.31	3.11 ± 1.57	4.89 ± 1.77
Female	48.4 ± 13.7	5.52 ± 2.05	2.57 ± 1.10	2.69 ± 1.42	5.23 ± 1.87
t-test (p-value)	0.3 (0.76)	0.1 (0.91)	0.3 (0.74)	2.10 (0.04)	1.4 (0.17)
**Age group** (in years)					
<30	47.2 ± 12.0	5.4 ± 1.2	2.4 ± 1.6	3.3 ± 2.0	4.5 ± 1.7
30-39	48.4 ± 16.1	5.5 ± 2.4	2.4 ± 1.3	2.8 ± 1.5	5.2 ± 1.9
40-49	49.9 ± 13.4	5.8 ± 1.8	2.4 ± 1.2	2.8 ± 1.6	5.4 ±1.5
50-59	50.6 ± 10.9	5.8 ± 1.4	2.7 ± 1.1	3.2 ± 1.5	5.1 ± 2.0
≥60	44.9 ± 10.5	4.6 ± 1.7	3.3 ± 0.8	3.0 ± 1.4	3.9 ± 1.7
F-test (p-value)	1.0 (0.39)	2.4 (0.05)	3.6 (0.01)	0.7 (0.62)	4.4 (0.00)
**Marital status**					
Married	49.3 ± 13.4	5.6 ± 1.89	2.7 ± 1.11	3.04 ± 1.59	4.93 ± 1.82
Unmarried	46.2 ± 11.4	5.24 ± 1.72	2.12 ± 1.67	2.41 ± 0.99	5.47 ± 1.75
t-test (p-value)	1.3 (0.20)	1.0 (0.30)	2.6 (0.01)	2.2 (0.03)	1.6 (0.11)
**Educational level**					
Primary or less	48.5 ± 10.4	5.5 ± 1.9	2.6 ± 1.2	2.9 ± 1.5	4.4 ± 2.1
At least secondary	48.8 ± 13.4	5.7 ± 1.6	2.7 ± 1.4	3.1 ± 1.3	5.1 ± 1.8
t-test (p-value)	0.1 (0.91)	0.3 (0.76)	0.6 (0.53)	0.5 (0.63)	1.4 (0.16)
**Occupation**					
Civil servant	46.9 ± 13.1	5.5±1.7	2.0 ± 1.3	2.4 ± 1.3	5.6 ± 1.7
Public servant	54.5 ± 13.4	6.2 ± 1.8	2.9 ± 0.7	3.9 ± 1.3	4.9 ± 2.0
Business/trader	50.3 ± 9.4	5.5 ± 1.8	3.3 ± 0.8	3.5 ± 1.4	4.3 ± 1.4
Others	39.4 ± 24.8	3.0 ± 2.7	3.5 ± 1.2	3.3 ± 2.4	3.3 ± 2.1
F-test (p-value)	4.8 (0.00)	7.1 (0.00)	22.3 (0.00)	14.8 (0.00)	10.8 (0.00)
**Respondent Source**					
Ward	47.1 ± 7.6	4.9 ± 1.6	3.7 ± 0.84	3.9 ± 0.58	3.11 ± 1.23
Clinic	49.0 ± 13.6	5.6 ± 1.9	2.5 ± 1.21	2.7 ± 1.56	5.20 ± 1.76
t-test (p-value)	0.6 (0.58)	1.5 (0.12)	4.0 (0.00)	2.8 (0.00)	4.9 (0.00)

**Table 6:** relationship between behavioural risk factors and knowledge of diabetes (N=212)

Variable	Total score Mean ± SD	Cause of DM Mean ± SD	Symptoms Mean ± SD	Complications Mean ± SD	Management Mean ± SD
**Consume alcohol**					
Yes	45.3 ± 13.3	5.3 ± 1.77	2.13 ± 1.59	2.29 ± 1.36	5.24 ± 1.91
No	50.7 ± 12.7	5.7 ± 1.91	2.87 ± 0.88	3.31 ± 1.49	4.90 ± 1.75
t-test (p-value)	2.9 (0.00)	1.5 (0.15)	4.4 (0.00)	4.9 (0.00)	1.3 (0.19)
**Smoke**					
Yes	51.5 ± 0.0	5.5 ± 5.77	3.5 ± 0.58	4.00 ± 1.16	4.00 ± 1.16
No	48.8 ± 13.3	5.5 ± 1.89	2.59 ± 1.23	2.92 ± 1.53	5.04 ± 1.82
t-test (p-value)	0.4 (0.68)	0.04 (0.97)	1.5 (0.14)	1.4 (0.16)	1.1 (0.26)
**Active physical exercise**					
Never	43.7 ± 14.0	5.3 ± 1.7	2.3 ± 1.5	2.2 ± 1.6	4.7 ± 1.9
Occasionally (<once a week)	47.1 ± 12.9	5.6 ± 2.0	2.3 ± 1.0	2.6 ±1.3	5.1 ± 1.9
Once or twice weekly	50.4 ± 11.8	5.5 ± 1.8	2.9 ± 1.3	3.1 ± 1.3	5.0 ± 1.4
Three or more times weekly	51.1 ± 11.8	5.9 ± 2.2	2.6 ± 1.2	3.4 ± 1.6	5.1 ± 1.6
F-test (p-value)	2.7 (0.045)	0.4 (0.75)	3.7 (0.01)	7.1 (0.00)	0.4 (0.79)
**Consume fresh fruits**					
Never	39.4 ± 15.3	5.0 ± 2.1	1.8 ± 1.3	2.2 ± 1.6	4.0 ± 1.9
Occasionally (<once a week)	47.4 ± 11.4	5.3 ± 1.9	2.5 ± 0.8	3.1 ± 1.3	4.8 ± 1.8
Once or twice weekly	49.5 ± 11.4	5.8 ± 1.6	2.8 ± 1.4	2.8 ± 1.5	4.9 ± 1.6
Three or more times weekly	52.3 ± 15.2	5.6 ± 2.0	2.7 ±1.3	3.2 ± 1.7	5.8 ± 1.9
F-test (p-value)	4.9 (0.00)	1.6 (0.20)	3.5 (0.02)	2.2(0.09)	5.9 (0.00)

## Discussion

Hypertension is a chronic disease that requires sustainable proactivity on the part of patients and caregivers towards the prevention of comorbidity. This study sought to assess the understanding of diabetes mellitus as a potential comorbidity among non-diabetic hypertensive patients in a developing country. Overall, respondents exhibited satisfactory knowledge of causes and risk factors, but an unsatisfactory understanding of signs and symptoms (Table 3and Table 4). This implies that hypertensive patients in a study setting know what may increase their risk of diabetes, but they may not recognise its onset and/or progression. Consequently, diabetes may begin and potentially progress while it remains unrecognised until irreversible comorbid complications present themselves [20]. This underscores the need for improved health education to address these knowledge gaps and encourage regular diabetes screening and preventive self-care measures among hypertensive patients in developing countries [21].

Older subjects in this study had significantly better knowledge of diabetes symptoms. Their heightened understanding may result from potentially better health literacy through extended experience with diabetic friends, relatives and other individuals who may have had observable clinical features of diabetes [22]. In view of the rising prevalence of diabetes, especially in families with higher genetic predispositions to cardiometabolic diseases, such health literacy is key [2,23]. Since subjects recruited from the wards had more comprehensive knowledge of diabetes symptoms and complications than those from the clinic, it is possible that a greater degree of formal and informal exposure to health education about cardiometabolic disease prevention can be achieved through prolonged interactions with healthcare workers in wards compared to clinics. Goal-oriented delivery of health information may be more attainable in wards where patients have more regular and measurable contact with healthcare providers [24,25]. For more holistic diabetes education among hypertensive patients, strategies that are better suited to less frequent contact with healthcare workers should be devised for outpatient clinic attendees [3,21].

In this study, there was no significant relationship between knowledge about diabetes causes and each of the assessed behavioural risk factors (p>0.05, Table 6). This suggests that preventive practices may not necessarily be influenced by understanding the benefits of diabetes risk reduction practices [26]. However, disparities were found in other aspects of diabetes knowledge; for example, fresh fruit consumption and frequent exercise were associated with greater knowledge of diabetes symptoms, complications and management. In other words, the motivation to practice preventive measures may result from knowledge of not diabetes causes, but diabetes symptoms and complications. This suggests that in the study setting, health education that outlines diabetes symptoms and complications could help improve the perception of diabetes disease features and severity, yielding better behavioural outcomes than materials focusing on causes and risk factors. In the study setting, diabetes disease severity may be more significant than susceptibility in motivating prevention, as enshrined in several psychological models of health behaviour [27].

Knowledge of diabetes is essential, especially among high risk individuals, but it does not always encourage diabetes prevention measures [26]. Many other intrinsic and extrinsic factors and complex interactions may be involved in determining decisions to commence and sustain preventive behaviours [27,28]. These include individual personalities, life experiences and expectations, an accessible environment for physical exercise and the availability and affordability of fresh fruit or non-alcoholic juices [27,29]. Unfortunately, many of these factors were not assessed in this study, which limited the possible associations found between knowledge and behavioural data. Therefore, causality may not be inferred from findings in this study. Nevertheless, the findings from this study contribute to the literature on hypertensive patients´ knowledge of different aspects of diabetes in a developing country setting.

## Conclusion

This study reveals that hypertensive patients´ knowledge of diabetes mellitus is generally lacking. Particularly poor knowledge of symptom recognition and diabetes complications may contribute to a high burden of undiagnosed diabetes in developing countries. The attainment of health-related Sustainable Development Goals (SDGs) requires improvement in the public´s knowledge and practice of preventive measures, especially among high-risk groups such as hypertensive patients. Healthcare providers and policymakers must therefore redouble their efforts in improving the general population´s systematic health education with a focus on high-risk groups. Further research in other developing countries is recommended, particularly qualitative studies that explore other relevant variables for a better understanding of the non-knowledge-related factors associated with the practice of preventive measures among hypertensive patients

### What is known about this topic

- Knowledge of diabetes is inadequate among individuals in general population, irrespective of level of risk of developing the disease;
- Hypertensive patients through regular clinic visit, are expected to have improved knowledge of cardiometabolic disease, including prevention of diabetes;
- Most patients who are already diabetic have improved knowledge of the disease prevention, especially through facility-based health education.

### What this study adds

- This study has identified inadequate knowledge of diabetes mellitus with focus on non-diabetic hypertensive patients as the study population;
- This study has also identified common areas of poor knowledge of diabetes that may be useful for prioritizing areas of emphasis during diabetes educational intervention;
- There is need for healthcare providers to improve on efforts aimed at providing effective diabetes education to hypertensive patients.

## References

[1] Manne-Goehler J, Atun R, Stokes A, Goehler A, Houinato D, Houehanou C (2016). Diabetes diagnosis and care in sub-Saharan Africa: pooled analysis of individual data from 12 countries. The lancet Diabetes & endocrinology.

[2] Cho N, Shaw J, Karuranga S, Huang Y, da-Rocha Fernandes, Ohlrogge A (2018). IDF Diabetes Atlas: Global estimates of diabetes prevalence for 2017 and projections for 2045. Diabetes research and clinical practice.

[3] Herman W (2017). The global agenda for the prevention of type 2 diabetes. Nutrition reviews.

[4] Pradeepa R (2013). The rising burden of diabetes and hypertension in southeast asian and african regions: need for effective strategies for prevention and control in primary health care settings. Int J Hypertens.

[5] Cheung B (2010). The hypertension-diabetes continuum. J Cardiovasc Pharmacol.

[6] Cheung B (2008). Hypertension as part of the metabolic syndrome. J Hum Hypertens.

[7] Leeson P (2017). Hypertension and cardiovascular risk in young adult life: insights from CAVI. European Heart Journal Supplements.

[8] Shen L, Shah B, Reyes E, Thomas L, Wojdyla D, Diem P (2013). Role of diuretics, β blockers, and statins in increasing the risk of diabetes in patients with impaired glucose tolerance: reanalysis of data from the NAVIGATOR study. BMJ.

[9] Rizos C, Elisaf M (2014). Antihypertensive drugs and glucose metabolism. World J Cardiol.

[10] Al-Mustafa B, Al-Ghareeb G, Wahabi H, Fayed AA (2018). Incidence and predictors of new onset type 2 diabetes among hypertensive-obese patients. Journal of Hypertension.

[11] Balogun WO, Balogun BL (2011). Co-occurrence of diabetes and hypertension: pattern and factors associated with order of diagnosis among Nigerians. Annals of Ibadan Postgraduate Medicine.

[12] Gorostidi M, de la Sierra A, González-Albarrán O, Segura J, de la Cruz JJ (2011). Abnormalities in ambulatory blood pressure monitoring in hypertensive patients with diabetes. Hypertension Research.

[13] Bruno RM, Penno G, Daniele G, Pucci L, Lucchesi D, Stea F (2012). Type 2 diabetes mellitus worsens arterial stiffness in hypertensive patients through endothelial dysfunction. Diabetologist.

[14] Ferrannini E, Cushman W Diabetes and hypertension: the bad companions. The Lancet. 2012;.

[15] Maina W, Ndegwa Z, Njenga E, Muchemi E (2010). Knowledge, attitude and practices related to diabetes among community members in four provinces in Kenya: a cross-sectional study. Pan Afr Med J.

[16] Odili V, Isiboge P, Eregie A (2011). Patients' Knowledge of diabetes mellitus in a Nigerian city. Tropical Journal of Pharmaceutical Research.

[17] Renzaho A (2015). The post-2015 development agenda for diabetes in sub-Saharan Africa: challenges and future directions. Global Health Action.

[18] Villagomez ET Health beliefs, knowledge, and metabolic control in diabetic Mexican American adults (Doctoral dissertation University of Texas Health Science Center at Houston. School of Nursing).

[19] Garcia AA, Villagomez ET, Brown SA, Kouzekanani K, Hanis CL (2001). The Starr County Diabetes Education Study: development of the Spanish-language diabetes knowledge questionnaire. Diabetes care.

[20] Kahn R, Alperin P, Eddy D, Borch-Johnsen K, Buse J, Feigelman J Age at initiation and frequency of screening to detect type 2 diabetes: a cost-effectiveness analysis. The Lancet. 2010;.

[21] Echouffo-Tcheugui J, Dagogo-Jack S (2012). Preventing diabetes mellitus in developing countries. Nature Reviews Endocrinology.

[22] Aboumatar H, Carson K, Beach M, Roter D, Cooper L (2013). The impact of health literacy on desire for participation in healthcare, medical visit communication, and patient reported outcomes among patients with hypertension. J Gen Intern Med.

[23] Hu F (2011). Globalization of diabetes: the role of diet, lifestyle, and genes. Diabetes Care.

[24] Sperl-Hillen J, Beaton S, Fernandes O, Von-Worley A, Vazquez-Benitez G, Parker E (2011). Comparative effectiveness of patient education methods for type 2 diabetes: a randomized controlled trial. Arch Intern Med.

[25] Khunti K, Gray L, Skinner T, Carey M, Realf K, Dallosso H (2012). Effectiveness of a diabetes education and self management programme (DESMOND) for people with newly diagnosed type 2 diabetes mellitus: three year follow-up of a cluster randomised controlled trial in primary care. BMJ.

[26] Wolde M, Berhe N, Van-Die I, Medhin G, Tsegaye A (2017). Knowledge and practice on prevention of diabetes mellitus among Diabetes mellitus family members, in suburban cities in Ethiopia. BMC research notes.

[27] Linke S, Robinson C, Pekmezi D (2014). Applying psychological theories to promote healthy lifestyles. American Journal of Lifestyle Medicine.

[28] Majumdar S, McAlister F, Furberg C (2004). From knowledge to practice in chronic cardiovascular disease: a long and winding road. J Am Coll Cardiol.

[29] Jack-Jr L (2005). Beyond lifestyle interventions in diabetes: a rationale for public and economic policies to intervene on social determinants of health. J Public Health Manag Pract.

